# Evaluation of antioxidant and antimicrobial activity of oregano (*Origanum vulgare* L.) preparations during storage of low-pressure mechanically separated meat (BAADER meat) from chickens

**DOI:** 10.1007/s10068-018-0491-1

**Published:** 2018-10-16

**Authors:** Elżbieta Hać-Szymańczuk, Aneta Cegiełka, Magdalena Karkos, Małgorzata Gniewosz, Kamil Piwowarek

**Affiliations:** 1 Department of Biotechnology, Microbiology and Food Evaluation, Faculty of Food Sciences, Warsaw University of Life Sciences—WULS SGGW, Nowoursynowska 159c, 02-787 Warsaw, Poland; 2 Department of Food Technology, Faculty of Food Sciences, Warsaw University of Life Sciences—WULS SGGW, Nowoursynowska 159c, 02-787 Warsaw, Poland

**Keywords:** Antimicrobial activity, Antioxidant activity, BAADER meat from chickens, Oregano, Storage

## Abstract

The goal of this study was to determine the effect of *Origanum vulgare* L. (oregano) preparations on the storage stability of vacuum-packed low-pressure mechanically separated meat (BAADER meat) from chickens stored at − 18 °C for 9 months. Oregano was added into the meat as a dried spice, extracts in water and ethanol (40 and 70% (*v/v*)), and an essential oil. The control samples did not contain oregano. The samples were examined immediately after arrival into the laboratory and after 1, 2, 3, 4, 6, and 9 months of storage. Oregano essential oil was most effective in slowing down lipid oxidation and inhibiting the growth of bacteria in BAADER meat. The number of coliform bacteria in the BAADER meat samples with the 0.1% essential oil was significantly lower than that in the control samples. The storage time was seen to have a more significant effect on the quality of BAADER meat.

## Introduction

Increased production of chickens for culinary purposes and manufacturing meat products has resulted in a higher yield of poultry skeletons, bones, and trimmings that remain after the largest muscles (breast and thigh) are separated. These parts still contain a significant part of muscle tissue, most of which can be recovered through mechanically separated meat (MSM) production (EFSA, 2013; Ruk, 2011). Production issues, quality requirements, and storage/use guidelines for MSM are regulated by the European Parliament. According to MSM definition in Commission Regulation No. 853/2004 (2004), mechanically separated poultry meat is a product obtained from poultry carcasses by removing meat from tissues adhering to the bones, using mechanical means, which leads to loss or modification of muscle fiber structure. The quality as well as the processing utility of this raw material largely depend on the technique through which it is obtained. MSM can be separated using high- or low-pressure technology, known in industrial practice as “high-pressure MSM” and “low-pressure MSM.” In the European poultry industry, high-pressure techniques are most often used to obtain MSM from chickens (EFSA, 2013). The raw material is characterized by a very high degree of disintegration, a high fat proportion, a paste-like consistency, and significant amounts of bone marrow from crushed bones. The low-pressure technique involves the use of devices that separate the remains of muscle tissue from the connective tissue and tendons without damaging the bone structure. A characteristic feature of low-pressure MSM is therefore the preservation of muscle fibre structure and significantly lower content of bone fragments, compared to the product obtained with high-pressure MSM (Botka-Petrak et al., 2011; Nagy et al., 2007).

One of the low-pressure MSM technologies currently used worldwide was developed by the BAADER company. BAADER meat is obtained using original Baader^®^ Food Processing Machinery equipped with a belt-drum system (EFSA, 2013). Due to the lesser degree of damage of muscle fibers and a smaller content of bone fragments as well as lower microbiological contamination, BAADER meat has a structure similar to ground meat and is considered to have better technological and processing usefulness than high-pressure MSM (EFSA, 2013; Henckel et al., 2004).

BAADER meat should be used in processing immediately after it is obtained. If this is not possible, this raw material can be stored in a frozen state (Ruk, 2011). The literature data indicate that, in addition to lower temperatures, plant-derived substances having antimicrobial and antioxidant properties are increasingly being used to prolong storage stability of poultry meat in various forms (Karre et al., 2013). However, the use of plant preparations to slow down adverse changes in the low-pressure quality of MSM has not been well researched.

In recent years, researchers and food producers have been increasingly studying oregano (*Origanum vulgare* L., family *Laminaceae*) (Karre et al., 2013). Oregano is added to dishes in the form of fresh and/or dried leaves, while water and alcohol extracts of oregano (Hernández-Hernández et al., 2009; Sánches-Escalante et al., 2003) and its essential oil (Chouliara et al., 2007; Fasseas et al., 2007) can also be used in food processing. The primary reason for numerous studies on the use of this spice in food is its high antioxidant (Kozłowska and Ścibisz, 2011; Sasse et al., 2009; Vallverdú-Queralt et al., 2014) and antimicrobial (Hać-Szymańczuk et al., 2014) activity. These properties of oregano have also been confirmed, inter alia, in studies on prolonging the shelf life of meat and meat products (Hernández-Hernández et al., 2009; Nieto et al., 2013; Rojas and Brewer, 2008; Sánchez-Escalante et al., 2003; Sasse et al., 2009; Shan et al., 2009).

The aim of this study was an evaluation of the antioxidant and antibacterial activity of oregano (*Origanum vulgare* L.) preparations in BAADER meat from chickens when storing it under frozen conditions. To the best of the authors’ knowledge, this is the first work on the preservation of this raw material with the use of plant preparations.

## Materials and methods

### Materials

The experimental material consisted of BAADER meat from chickens obtained by using the desinewing technology developed by the Baader^®^ Food Processing Machinery company. The belt-drum separator BAADER 605 (Baader^®^ Food Processing Machinery, Trige, Denmark)—situated in a production plant located in northeast Poland—was used to separate meat from connective tissue and sinews. The raw materials for BAADER meat were chicken broilers’ femurs and scraps of thigh muscles, which had been obtained immediately after manual removal of thigh muscles.

The chilled BAADER meat, sourced from four production batches (weight of each batch: 6 kg) was transported to the Department of Biotechnology, Microbiology and Food Evaluation of the Warsaw University of Life Sciences (WULS–SGGW) under refrigerated conditions. Oregano (*Origanum vulgare* L.) was added to the BAADER meat in dried form (“Kamis^®^”, McCormic, Stefanowo, Poland) and as a water extract, ethanol extract, and essential oil (prepared by the authors themselves) under laboratory conditions. These are subsequently referred to as oregano preparations.

The water and ethanol extract of dried oregano were obtained via continuous extraction in a Soxhlet apparatus (Universal Extraction System B-811, Büchi Labortechnik AG, Flawil, Switzerland). The parameters of the extraction process were established in the preliminary study (results unpublished). To prepare each extract, 40 g of dried oregano was distributed into 8 extraction thimbles (5 g per thimble). Distilled water and ethyl alcohol with 40 and 70% (*v/v*) concentration were used as solvents. The raw material in each thimble was extracted with 150 mL of the appropriate solvent for 15 cycles while maintaining the solvent boiling point. The portions obtained from each extract were combined, resulting in approximately 550 mL of raw extracts. The raw extracts were filtered using 180-μm thick filter paper (Whatman GE, LaboPlus Sp. z o.o., Warsaw, Poland). Subsequently, each extract was concentrated in a rotary evaporator Model R-205 (Büchi Labortechnik AG) until approximately 40 g of the extract was left, corresponding to the weight of dried oregano used to obtain the extract.

To obtain essential oil from oregano, the method described by Białecka-Floriańczyk and Włostowska (2007) was followed. Around 30 g of fresh oregano leaves were crumbled, covered with 400 mL of water, and subjected to distillation in a Deryng apparatus (Simax® KAVALIERGLASS, A.S., Prague, Czech Republic) until essential oils were obtained. The chilled distillate was extracted four times using dichloromethane in a separatory funnel, after which water was removed by adding anhydrous magnesium sulfate. The obtained extract was concentrated in a Büchi rotary evaporator model R-205. The solvent was evaporated at a temperature of 30 °C and pressure 540–560 hPa.

The characteristic chemical compounds of the oregano preparations were identified and their quantitative amounts determined. For the qualitative and quantitative composition analysis of the oregano extracts, high-performance liquid chromatography (HPLC) was applied using Agilent 1200 liquid chromatograph (Agilent^®^ Technologies, Santa Clara, CA, USA) coupled with a diode array detector (DAD). The determination of volatile compounds in oregano essential oil was performed with a AutoSystem XL Gas Chromatograph (PerkinElmer Inc., Waltham, MA, USA) equipped with a flame ionization detector (FID). The equipment of the chromatographs and the process conditions were described by Hać-Szymańczuk et al. (2017).

Each of the four production batches of BAADER meat from chickens was analyzed to determine the basic chemical composition. The fat, protein, and water levels were determined using a FoodScan™Lab near-infrared spectrometer (Foss Analytical A/S, Hillerød, Denmark), operating in the spectral range 850–1050 nm and using a calibration based on the artificial neural network model (AOAC, 2007). Portions of BAADER meat (250 g) were placed on a plastic dish. The thickness of the sample layer was 0.5 cm. The measurement was conducted in three repetitions for each sample.

### Performance of the experiment

In each of the four experimental series, six samples of BAADER meat were prepared (each weighing 1.5 kg), differing in terms of the type of oregano preparation added: Control: sample without addition of oregano, D: 2.0% addition of dried oregano, WE: 2.0% addition of water extract from oregano, E40: 2.0% addition of 40% (*v/v*) ethanol extract from oregano, E70: 2.0% addition of 70% (*v/v*) ethanol extract from oregano, and EOS: 0.1% addition of essential oil from oregano. The addition level of the oregano preparations to the BAADER meat was established based on the literature (Hać-Szymańczuk et al., 2017). After the samples were thoroughly mixed with oregano preparations, each sample was divided into six portions (250 g each), which were vacuum-packed in plastic bags (PE/PA, thickness 75 µm) using a vacuum machine (C200, Multivac Sepp Haggenmüller GmbH & Co. K.G., Wolfertschwenden, Germany). These samples were stored at − 18 °C for 9 months.

After 0, 1, 2, 3, 4, 6, and 9 months of storage, the values of thiobarbituric acid reactive substances (TBARS) were determined and microbial analyses were performed twice for each sample. Before the analyses, except for “zero” time, each sample was defrosted (+ 4 °C, 4 h) without opening the packaging.

TBARS values were assayed using the extraction method described by Pikul et al. (1989). Absorbance was measured at 532 nm in a spectrophotometer (GENESYS™ 30 Visible Spectrophotometer, Thermo Fisher Scientific, Waltham, MA, USA). As a control, a mixture of 5 mL of 4% perchloric acid and 5 mL of a 0.02 M solution of 2-thiobarbituric acid was used. The TBARS values were expressed as milligrams of malondialdehyde (MAD) per kilogram of BAADER meat. The TBARS values were calculated according to the formula:

$${\text{TBARS}} = {\text{K}}{ \times }{\text{E,}}$$

where K is the conversion factor of 5.5, and E represents the absorbance value.The microbial analyses were conducted following Polish Standards. The foil packaging containing meat was opened using a sterile scalpel. From the packaging, 20 g of the sample was taken using a sterile spoon and transferred to 180 mL of 0.1% sterile peptone water (bioMérieux, Paris, France). Each sample was homogenized in a Stomacher blender (Lab Blender 400 Circulator, Seward Laboratory, London, UK) for 1 min at high speed and at room temperature (18 °C). Further, a series of 10-fold dilutions in sterile peptone water were prepared.

The microbial analyses included the determination of the total bacterial count (TBC; 30 °C for 48 h) and the number of psychrotrophic bacteria (6 °C for 10 days) on plate count agar (BTL, Lodz, Poland), *Enterobacteriaceae* (37 °C for 24 h) on violet red bile glucose agar (BTL), coliform bacteria (37 °C for 48 h) on Endo agar (Biomed, Krakow, Poland), and *Salmonella* spp. The presence of *Salmonella* spp. in 25 g of product was determined using Müller-Kauffman’s medium with tetrathionate and novobiocin, RVS (medium according to Rappaport-Vassilliads with soya), xylose lysine deoxycholate, and Hektoen (BTL). Colonies typical for *Salmonella* spp. and suspicious colonies were confirmed using API 20E biochemical tests (bioMerieux). The number of bacteria was expressed as log_10_ of colony-forming units per gram of meat (log CFU/g).

The experiment was repeated four times. Statistical analysis of the results was performed using Statistica ver. 10.0 software (StatSoft Inc., Tulsa, OK, USA). The significance was tested using one-way analysis of variance (ANOVA) and Tukey’s honest significant difference (HSD) test, at a significance level of *α* = 0.05.

## Results and discussion

The fat, protein, and water content in BAADER meat obtained from chicken thigh scraps was 11.78, 18.54, and 69.21%, respectively.

The literature indicates that the chemical composition of MSM from chickens fluctuates, as it depends, inter alia, on the production method and the machine used as well as the parts of deboned carcass (Bełkot et al., 2013; Botka-Petrak et al., 2011). Henckel et al. (2004) found that the fat, protein, and water content of low-pressure MSM made from chicken breast muscles was 10.35, 18.78, and 70.27%, which is similar to the results seen in our study.

The results obtained indicate that oregano preparations used in this study differed in terms of the type and quantity of active substances (Table 1). In the oregano extract dominated rosemary acid, *p*-coumaric acid, chlorogenic acid, and carnosol. The acids were also the main components of the oregano ethanol extracts. It was also found that the 40% ethanol extract (*v/v*) of oregano contained over twice as much carnosol as the 70% extract. In turn, the essential oil obtained from this plant in terms of quantity was dominated by: carvacrol, 1,4-cineole, *γ*-terpinene, thymol, and *β*-caryophyllene.

**Table 1:** Selected chemical compounds and their content in oregano extracts and essential oil

Chemical compound	Retention time	Water extract	Ethanol 40% v/v extract	Ethanol 70% v/v extract
Water and ethanol extracts of oregano				
Chlorogenic acid	2.83^1^	0.263^2^	0.714^2^	0.386^2^
Epicatechin	3.80	ND	0.006	0.004
Caffeic acid	4.33	0.012	0.044	0.020
*p*-coumaric acid	7.04	1.065	0.480	0.592
Ferrulic acid	8.08	ND	ND	0.010
Benzoic acid	11.87	0.075	0.076	0.086
Rosemary acid	12.70	1.824	2.344	2.836
Myricetin	12.80	0.170	0.046	0.060
Resveratrol	15.51	0.012	0.060	0.080
Quercetin	18.69	0.016	0.030	0.002
Carnosol	25.73	0.246	0.168	0.060
Curcumin	26.03	ND	ND	0.020

Chemical compound	Retention time	Essential oil
Oregano essential oil		
*α*-pinene	7.88^1^	0.06^2^
*β*-pinene	8.79	0.25
Myrcene	9.21	0.37
1.4-cineole	9.72	1.34
*p*-cymene	9.92	0.28
γ-terpinene	10.71	1.33
Linalol	11.70	0.54
Camphor	12.26	0.30
Borneol	13.15	0.33
Carvone	14.93	0.32
Thymol	15.99	0.94
Carvacrol	16.20	650.12
Eugenol	17.42	0.16
*β*-caryophyllene	18.73	0.86

^1^min

^2^mg/mL

ND—not detected

The results obtained confirmed that oregano preparations are constituted of complexes of many chemical compounds (Vallverdú-Queralt et al., 2014). The chemical compounds mentioned by the authors, although in different amounts, were also identified by other researchers in oregano essential oil (dos Santos Rodrigues et al., 2017) and oregano extract (Kocić-Tanackov et al., 2012).

The antioxidant properties of oregano are due to the presence of phenolic acids, including caffeic, neochlorogenic, rosmarinic, and carnosic acid, and carnosol (Hernández-Hernández et al., 2009). The antioxidant activity of oregano is also attributed to the presence of carvacrol, thymol, and *p*-cymene, all of which chelate metal ions and react with free radicals (Kozłowska and Ścibisz, 2011). According to Capecka et al. (2005), the antioxidant activity of plant extracts depends not only on the number of polyphenolic compounds, but also on their chemical structure. The large ability to scavenge free radicals is demonstrated, inter alia, by rosmarinic acid contained in oregano, due to hydroxyl groups that are present in the ring in the *ortho*-position.

The antimicrobial activity of oregano is associated with the presence of components such as phenols, alcohols, ketones, ethers, and hydrocarbons (Grochev and Girow, 2009; Kozłowska and Ścibisz, 2011). According to Grochev and Girow (2009), the active compounds thymol and carvacrol have the strongest antimicrobial activity in oregano oil. The actions of these compounds on bacterial cells has been recognized. In relation to Gram-negative bacteria, this mechanism comprises disintegration of the cell membrane by interference with the structure of lipopolysaccharides and increasing the permeability of cytoplasmic membrane to ATP, the leak of which eventually leads to cell death (Helander et al., 1998). In the case of Gram-positive bacteria, carvacrol interacts with the cell membrane, changing its permeability to H^+^ and K^+^ cations. The change in the gradient of these cations disturbs the basic processes in the cell, resulting in its death (Ultee et al., 2002).

The progress of the oxidation of lipids in foods can be measured by determination of secondary products of this process. One of them is malondialdehyde (MDA) which presence in meat is mainly associated with off-flavors. One of numerous methods for measuring MDA is through reaction of it with thiobarbituric acid (TBA) to produce a pink-colored compound. The substances that also react with TBA but which are not MDA, are called TBA-reactive substances (TBARS) (Pikul et al., 1989).

Based on the TBARS index, it was determined that the oregano essential oil had the most effective action in limiting lipid oxidation in BAADER meat during 9 months of storage (Table 2). The initial TBARS index value for BAADER meat was 0.306 mg MAD/kg of the sample. Compared to EOS, significantly (*p* < 0.05) higher TBARS values were found in D and E40. After 9 months of sample storage, the TBARS index for EOS was 0.118 mg MAD/kg of the sample and was significantly (*p* < 0.05) lower than that seen in the other samples.

**Table 2:** Effect of addition of oregano preparations on TBARS index value in BAADER meat from chickens stored at − 18 °C

Storage time (month)	Control sample	Dried oregano	Water extract	Ethanol 40% *v/v* extract	Ethanol 70% *v/v* extract	Essential oil
0	0.306^1^ ± 0.008^2^ABa	0.306 ± 0.008Ba	0.306 ± 0.008ABa	0.306 ± 0.008Ba	0.306 ± 0.008ABa	0.306 ± 0.008Aa
1	0.326 ± 0.060ABab	0.558 ± 0.011Bab	0.444 ± 0.132ABab	0.404 ± 0.172Bab	0.439 ± 0.127ABab	0.307 ± 0.118Aab
2	0.350 ± 0.017ABb	0.514 ± 0.160Bb	0.532 ± 0.033ABb	0.504 ± 0.219Bb	0.394 ± 0.102ABb	0.342 ± 0.143Ab
3	0.362 ± 0.042ABa	0.341 ± 0.070Ba	0.290 ± 0.055ABa	0.244 ± 0.080Ba	0.270 ± 0.080ABa	0.297 ± 0.026Aa
4	0.435 ± 0.030ABab	0.409 ± 0.090Bab	0.319 ± 0.014ABab	0.354 ± 0.055Bab	0.293 ± 0.035ABab	0.257 ± 0.036Aab
6	0.425 ± 0.021ABb	0.586 ± 0.032Bb	0.355 ± 0.169ABb	0.528 ± 0.022Bb	0.392 ± 0.018ABb	0.385 ± 0.090Ab
9	0.492 ± 0.031ABab	0.485 ± 0.063Bab	0.347 ± 0.012ABab	0.355 ± 0.095Bab	0.227 ± 0.089ABab	0.118 ± 0.022Aab

A, B means with different capital letters in the same row are significantly different (*p *< 0.05)

a, b means with different lowercase letters in the same column are significantly different (*p *< 0.05)

^1^mg MAD/kg of meat

^2^standard deviation

No information was found in the literature regarding the use of oregano preparations to improve the oxidative stability of BAADER meat. Most studies concern poultry meat (Botsoglou et al., 2003; Sampaio et al., 2012) or mammals (Fasseas et al., 2007; Rojas and Brewer, 2008; Shan et al., 2009) and products made from them (Hernández-Hernández et al., 2009; Nieto et al., 2013; Sánchez-Escalante et al., 2003).

Based on the results of several different tests used to assess the lipid oxidation status, it was shown that cooked breast and thigh muscles of chickens previously treated with marinade containing dried oregano, sage, and honey showed higher oxidative stability than unmarinated meat after 96 h storage at 4 °C (Sampaio et al., 2012). A combination of adding oregano preparation and the appropriate packaging method results in a good effect in terms of prolonging the duration of the suitability of poultry meat for consumption. In case of breast muscles of chickens with the addition of oregano oil packed in MAP packaging, the oxidation and microbiological changes were slowed down, which resulted in prolonging their shelf life by 5–6 days (Chouliara et al., 2007).

The antioxidant effect of water oregano extracts was demonstrated in storage stability studies on vacuum-packed raw beef and pork patties that were stored frozen for 4 months (Rojas and Brewer, 2008). However, the oxidative and microbiological stability of raw ground pork (Hernández-Hernández et al., 2009; Nieto et al., 2013; Shan et al., 2009) and beef patties (Sánchez-Escalante et al., 2003) improved on using alcohol oregano extracts. Assessing the storage stability of pork and beef after the addition of plant extracts, it was also seen that the antioxidant effect of oregano is more effective in heat-treated than in raw meat (Fasseas et al., 2007).

On the basis of the obtained results, a tendency to limit the growth of mesophilic aerobic bacteria (TBC, Table 3) and psychrotrophic bacteria (Table 4) in BAADER meat from chickens with the addition of oregano preparations was observed. Compared to the Control sample, despite the statistically insignificant (*p* > 0.05) difference, the lowest number of these groups of microorganisms was found in the BAADER meat samples with added essential oil (EOS). With the storage time of BAADER meat in a frozen state, regardless of the type of oregano preparation, the number of TBC increased gradually, so that after 3 months, the statistically highest values (*p* < 0.05) were obtained. In turn, the number of psychrotrophic microorganisms in BAADER meat was the highest (*p* < 0.05) after 2 months of storage.

**Table 3:** Effect of addition of oregano preparations on the total bacteria count in BAADER meat from chickens stored at − 18 °C

Storage time (month)	Control sample	Dried oregano	Water extract	Ethanol 40% *v/v* extract	Ethanol 70% *v/v* extract	Essential oil
0	4.2^1^ ± 0.13^2^ Aa	4.2 ± 0.13Aa	4.2 ± 0.13Aa	4.2 ± 0.13Aa	4.2 ± 0.13Aa	4.2 ± 0.13Aa
1	4.3 ± 0.24Aa	4.7 ± 0.15Aa	4.6 ± 0.08Aa	3.7 ± 0.40Aa	4.3 ± 0.17Aa	4.2 ± 0.17Aa
2	5.7 ± 0.45Aa	5.5 ± 0.40Aa	5.5 ± 0.21Aa	5.3 ± 0.35Aa	5.5 ± 0.25Aa	5.2 ± 0.31Aa
3	6.2 ± 0.23Ab	5.3 ± 0.47Ab	6.0 ± 0.33Ab	5.5 ± 0.28Ab	5.7 ± 0.25Ab	5.6 ± 0.28Ab
4	6.5 ± 0.03Aab	6.1 ± 0.43Aab	5.5 ± 0.42Aab	5.4 ± 0.16Aab	5.4 ± 0.31Aab	5.4 ± 0.33Aab
6	3.6 ± 0.31Aa	3.4 ± 0.18Aa	3.4 ± 0.34Aa	2.8 ± 0.17Aa	3.3 ± 0.37Aa	3.0 ± 0.35Aa
9	3.7 ± 0.51Aa	3.1 ± 0.34Aa	3.2 ± 0.31Aa	3.2 ± 0.25Aa	3.5 ± 0.35Aa	3.0 ± 0.16Aa

A means with the same capital letter in the same row are not significantly different (*p *> 0.05)

a, b means with different lowercase letters in the same column are significantly different (*p *< 0.05)

^1^log CFU/g of meat

^2^standard deviation

**Table 4:** Effect of addition of oregano preparations on the number of psychrotrophic bacteria in BAADER meat from chickens stored at − 18 °C

Storage time (month)	Control sample	Dried oregano	Water extract	Ethanol 40% *v/v* extract	Ethanol 70% *v/v* extract	Essential oil
0	3.6^1^ ± 0.23^2^Aa	3.6 ± 0.23Aa	3.6 ± 0.23Aa	3.6 ± 0.23Aa	3.6 ± 0.23Aa	3.6 ± 0.23Aa
1	3.9 ± 0.23Aa	4.4 ± 0.18Aa	4.0 ± 0.17Aa	4.0 ± 0.40Aa	4.2 ± 0.35Aa	3.5 ± 0.43Aa
2	4.4 ± 0.16Ab	4.7 ± 0.31Ab	4.5 ± 0.28Ab	4.4 ± 0.30Ab	4.4 ± 0.16Ab	4.4 ± 0.51Ab
3	4.6 ± 0.35Aa	3.9 ± 0.28Aa	3.6 ± 0.31Aa	3.8 ± 0.51Aa	4.1 ± 0.25Aa	3.6 ± 0.33Aa
4	4.7 ± 0.33Aa	4.3 ± 0.23Aa	3.4 ± 0.43Aa	4.1 ± 0.14Aa	4.4 ± 0.31Aa	3.5 ± 0.40Aa
6	3.5 ± 0.25Aa	3.4 ± 0.40Aa	3.4 ± 0.33Aa	3.4 ± 0.35Aa	3.5 ± 0.28Aa	3.4 ± 0.23Aa
9	3.5 ± 0.17Aa	3.4 ± 0.51Aa	2.9 ± 0.16Aa	2.6 ± 0.31Aa	3.5 ± 0.17Aa	3.3 ± 0.18Aa

A means with the same capital letter in the same row are not significantly different (*p *> 0.05)

a, b means with different lowercase letters in the same column are significantly different (*p *< 0.05)

^1^log CFU/g of meat

^2^standard deviation

Addition of each of the oregano preparations also slightly limited the growth of bacteria from the *Enterobacteriaceae* family in BAADER meat (Table 5). The most effective in this respect was the essential oil (EOS) and 70% ethanol extract (E70). In all BAADER meat samples, significantly (*p* < 0.05) the highest number of *Enterobacteriaceae* bacteria was determined after 2 months of storage. Extending the storage period to 6 and 9 months resulted in a significant (*p* < 0.05) reduction in the number of these microorganisms.

**Table 5:** Effect of addition of oregano preparations on the number of *Enterobacteriaceae* bacteria in BAADER meat from chickens stored at − 18 °C

Storage time (month)	Control sample	Dried oregano	Water extract	Ethanol 40% *v/v* extract	Ethanol 70% *v/v* extract	Essential oil
0	3.4^1^ ± 0.26^2^Ab	3.4 ± 0.26Ab	3.4 ± 0.26Ab	3.4 ± 0.26Ab	3.4 ± 0.26Ab	3.4 ± 0.26Ab
1	3.6 ± 0.51Abc	3.2 ± 0.35Abc	3.6 ± 0.43Abc	3.5 ± 0.37Abc	3.5 ± 0.33Abc	3.5 ± 0.23Abc
2	3.9 ± 0.43Ac	3.8 ± 0.33Ac	3.7 ± 0.31Ac	3.7 ± 0.25Ac	3.7 ± 0.40Ac	2.8 ± 0.18Ac
3	3.9 ± 0.31Abc	3.7 ± 0.23Abc	2.6 ± 0.28Abc	3.5 ± 0.28Abc	2.8 ± 0.23Abc	2.4 ± 0.35Abc
4	3.9 ± 0.40Abc	3.5 ± 0.51Abc	3.7 ± 0.25Abc	2.6 ± 0.17Abc	3.3 ± 0.31Abc	2.4 ± 0.28Abc
6	1.4 ± 0.25Aa	1.2 ± 0.18Aa	1.5 ± 0.23Aa	1.5 ± 0.35Aa	1.5 ± 0.51Aa	1.1 ± 0.16Aa
9	1.4 ± 0.16Aa	1.5 ± 0.40Aa	1.0 ± 0.33Aa	1.7 ± 0.18Aa	1.4 ± 0.43Aa	1.0 ± 0.21Aa

A means with the same capital letter in the same row are not significantly different (*p *> 0.05)

a, b means with different lowercase letters in the same column are significantly different (*p *< 0.05)

^1^log CFU/g of meat

^2^standard deviation

The essential oil (EOS) showed the highest effectiveness in inhibiting the growth of coliform bacteria. The use of the essential oil resulted in a significant (*p* < 0.05) reduction in the number of these microorganisms compared to the Control (Table 6). BAADER meat analyzed after 6 and 9 months of storage and immediately after production (“0”) and 1 month of storage proved to be the least contaminated with these microorganisms.

**Table 6:** Effect of addition of oregano preparations on the number of coliform bacteria in BAADER meat from chickens stored at − 18 °C

Storage time (month)	Control sample	Dried oregano	Water extract	Ethanol 40% *v/v* extract	Ethanol 70% *v/v* extract	Essential oil
0	2.3^1^ ± 0.24^2^ Ba	2.3 ± 0.24ABa	2.3 ± 0.24ABa	2.3 ± 0.24ABa	2.3 ± 0.24ABa	2.3 ± 0.24Aa
1	3.8 ± 0.16Ba	3.2 ± 0.38ABa	3.1 ± 0.25ABa	2.5 ± 0.35ABa	3.4 ± 0.19ABa	3.2 ± 0.16Aa
2	3.8 ± 0.28Bb	3.7 ± 0.23ABb	3.4 ± 0.51ABb	3.4 ± 0.43ABb	3.7 ± 0.17ABb	3.3 ± 0.43Ab
3	3.8 ± 0.31Bb	3.2 ± 0.35ABb	3.5 ± 0.40ABb	3.5 ± 0.31ABb	3.6 ± 0.33ABb	3.3 ± 0.40Ab
4	3.8 ± 0.33Bb	3.8 ± 0.18ABb	3.4 ± 0.28ABb	3.4 ± 0.23ABb	2.7 ± 0.25ABb	2.6 ± 0.35Ab
6	1.5 ± 0.51Ba	1.3 ± 0.31ABa	1.0 ± 0.16ABa	1.4 ± 0.51ABa	1.0 ± 0.38ABa	1.4 ± 0.31Aa
9	1.6 ± 0.18Ba	1.0 ± 0.43ABa	1.0 ± 0.17ABa	1.0 ± 0.18ABa	1.0 ± 0.40ABa	1.0 ± 0.28Aa

A, B means with different capital letters in the same row are significantly different (*p *< 0.05)

a, b means with different lowercase letters in the same column are significantly different (*p *< 0.05)

^1^log CFU/g of meat

^2^standard deviation

No *Salmonella* spp. bacteria presence was found in 25 g of the examined BAADER meat samples. The raw material used in this study meets the food safety criteria for the presence of *Salmonella* and the hygiene criteria for minced meat for aerobic bacteria and *Escherichia (E.) coli*, as specified in Commission Regulation (2007).

It was found in storage test of turkey BAADER meat stored at − 18 °C for 12 months, that the microbiological quality of this raw material at the end of the storage period was satisfactory despite the negative organoleptic changes. The number of microorganisms determined in it after 9 months was: TBC 1,2 × 10^4^ CFU/g, *E. coli* < 10^2^ CFU/g, *Staphylococcus (S.) aureus* < 10^2^ CFU/g, sulfite reducing bacteria < 10^2^ CFU/g, *Salmonella spp.* not present in 25 g and *Listeria monocytogenes* < 10 CFU/g (Ruk,^2011^). In the light of the available knowledge, the current uses of the addition of oregano preparations did not concern BAADER meat. Knowledge of the properties of these preparations, resulting from the presence of many different antioxidant and antimicrobial substances, suggests that they may constitute an additional factor limiting adverse changes in quality of the examined raw material.

Depending on the form of the spices used (fresh leaves and fruits, essential oils, water and ethanol extracts, droughts), they exhibit diverse effects on the inhibition of microorganism growth in food. Burt (2004) reports, that the best antimicrobial properties are found in essential oils, fat extracts, and ethanol extracts of spices, while plant droughts and water extracts show a lower efficacy. The above thesis was confirmed with regard to oregano preparations, both in this work and in previous studies (Hać-Szymańczuk et al. 2014; 2015). According to the authors, this is due to the formation of different profiles of chemical compounds in these preparations. In the assessment of antimicrobial activity of oregano preparations, it was shown that Gram-positive bacteria (*Micrococcus* sp., *Bacillus subtilis*, *Tetracoccus* sp., *Staphylococcus aureus*, *Enterococcus faecalis*) were more sensitive to the essential oil (Hać-Szymańczuk et al., 2014) and water extract (Hać-Szymańczuk et al., 2015) of oregano than Gram-negative ones (*Proteus vulgaris*, *Proteus mirabilis*, *E. coli*, *Klebsiella (K.) pneumoniae*, *Salmonella* Enteritidis). The most resistant among the bacteria tested for the active components contained in the oregano oil were *E. coli* bacteria (Hać-Szymańczuk et al., 2014), and the lowest sensitivity to the activity of water extract from this plant was demonstrated by *K. pneumoniae* and *Salmonella Enteritidis* (Hać-Szymańczuk et al., 2015). The authors concluded that the antimicrobial activity of oregano oil was associated with the presence of compounds identified in it: carvacrol, camphor, linalol, R(+) limonene, 1,4-cineol and *γ*-terpinene, and in the case of extract: chlorogenic and *p*-coumarin acid as well as mirycetin.

In order to prolong the shelf life of chicken meat using oregano preparations, combined preservation methods may be used. In the studies on the influence of water oregano extract and packaging method on the microbiological quality of chicken carcasses stored at 4 °C was found, that the use of vacuum packaging resulted in a reduction of microbiological contamination and elongation of shelf life compared to carcasses that were air-packaged on trays (Khaled et al., 2016). Chouliara et al. (2007) used 0.1% oregano oil and MAP packaging and obtained a slowdown in microbiological changes that occurred due to reduction in the TBC, *Pseudomonas* spp., lactic acid bacteria, *Brochothrix thermosphacta, Enterobacteriaceae*, and yeast in chicken breast muscles. However, the results of antimicrobial activity of oregano preparations obtained in the model system are not always transferable to the food matrix.

The results obtained in this study indicate that the addition of oregano preparations may be an auxiliary factor in prolonging the storage stability of vacuum-packed BAADER meat from chickens stored frozen for 9 months. The highest activity in inhibiting oxidative changes of lipids and limiting the growth of microflora in BAADER meat from chickens was demonstrated for oregano essential oil.
